# A Combined Western and Bead-Based Multiplex Platform to Characterize Extracellular Vesicles

**DOI:** 10.1089/ten.tec.2023.0056

**Published:** 2023-11-06

**Authors:** Josette C. van Maanen, Frances C. Bach, Theresa S. Braun, Alberta Giovanazzi, Bas W.M. van Balkom, Markus Templin, Marca H.M. Wauben, Marianna A. Tryfonidou

**Affiliations:** ^1^ Department of Clinical Sciences, Faculty of Veterinary Medicine, Utrecht University, Utrecht, the Netherlands.; ^2^ NMI Natural and Medical Sciences Institute at the University of Tübingen, Reutlingen, Germany.; ^3^ Department of Biomolecular Health Sciences, Faculty of Veterinary Medicine, Utrecht University, Utrecht, the Netherlands.; ^4^ Department of Nephrology and Hypertension, University Medical Center Utrecht, Utrecht, the Netherlands.; ^5^ NMI TT Pharmaservices, Berlin, Germany.

**Keywords:** DigiWest technology, extracellular vesicles, protein markers, multiplex characterization

## Abstract

In regenerative medicine, extracellular vesicles (EVs) are considered as a promising cell-free approach. EVs are lipid bilayer-enclosed vesicles secreted by cells and are key players in intercellular communication. EV-based therapeutic approaches have unique advantages over the use of cell-based therapies, such as a high biological, but low immunogenic and tumorigenic potential. To analyze the purity and biochemical composition of EV preparations, the International Society for Extracellular Vesicles (ISEV) has prepared guidelines recommending the analysis of multiple (EV) markers, as well as proteins coisolated/recovered with EVs. Traditional methods for EV characterization, such as Western blotting, require a relatively high EV sample/protein input for the analysis of one protein. We here evaluate a combined Western and bead-based multiplex platform, called DigiWest, for its ability to detect simultaneously multiple EV markers in an EV-containing sample with inherent low protein input. DigiWest analysis was performed on EVs from various sources and species, including mesenchymal stromal cells, notochordal cells, and milk, from human, pig, and dog. The study established a panel of nine antibodies that can be used as cross-species for the detection of general EV markers and coisolates in accordance with the ISEV guidelines. This optimized panel facilitates the parallel evaluation of EV-containing samples, allowing for a comprehensive characterization and assessment of their purity. The total protein input for marker analysis with DigiWest was 1 μg for all nine antibodies, compared with ∼10 μg protein input required for traditional Western blotting for one antibody. These findings demonstrate the potential of the DigiWest technique for characterizing various types of EVs in the regenerative medicine field.

**Impact statement:**

In regenerative medicine, extracellular vesicles (EVs) have gained attention as a cell-free approach because of their high biological and low immunogenic/tumorigenic potential. We here explored a multiplex bead-based Western blot technique (DigiWest) for the characterization of EV preparations from different sources and species with regenerative potential. Using this technology, which can simultaneously detect many different proteins in a single EV sample and requires low total protein input, we established a panel of nine antibodies that can be used for cross-species EV preparations analysis. As such, the DigiWest technology might facilitate the translation of EV-based regenerative approaches.

## Introduction

Within the field of cell-based therapies, different approaches apply, such as the transplantation of tissue-specific cells^1^ or the application of stem cells (e.g., mesenchymal stromal cells [MSCs]^2^). Their clinical application is, however, limited due to the complicated regulatory pathways for cell therapies^3^ and the inherent high costs.^4^ To overcome this, universal donor-derived cells (e.g., allogenic)^5^ can be used, but as major drawback this can elicit an immune response in the patient. Although several strategies are developed to reduce these responses, none successfully ensure long-term survival of grafted cells.^6^

A promising cell-free alternative is the use of extracellular vesicles (EVs).^7^ EVs are lipid bilayer-enclosed vesicles secreted by cells under both physiological and pathological conditions.^8–10^ They play an important role in intercellular signaling, thereby influencing the behavior of target cells (e.g., cell phenotype, proliferation, and differentiation).^7^ EVs in regenerative medicine are of special interest, since EVs derived from regenerative stem cell populations harness a similar biological activity as the parent cell, but are considered to have no tumorigenic potential.^7,11^ The immunogenicity of EVs is, in contrast to that of cells, considered to be minimal, even when they are added from an allogenic source.^12–14^ Altogether, these unique advantages make EVs an attractive alternative to cell therapies.

Before EVs can be applied in a clinical setting, there are several bottlenecks with regard to the biochemical composition and the purity of EV preparations.^15^ EVs are commonly isolated from biological fluids or conditioned culture medium (CM).^16^ Besides a wide plethora of different EV types, these sources contain other colloidal structures such as cells, proteins, and lipoprotein particles.^17,18^ To assess the purity of EV preparations and to ensure that the observed effects are EV-mediated, characterization of EV samples is essential.^18,19^

The International Society for Extracellular Vesicles (ISEV) has published guidelines for EV characterization recommending a list of protein markers to demonstrate the lipid bilayer structure of EVs (e.g., transmembrane or glycosylphosphatidylinositol-anchored proteins) and to identify luminal protein cargo (e.g., soluble cytosolic proteins). Furthermore, suggestions are made to analyze proteins that might be coisolated/recovered with EVs.^19^

The most commonly used method for characterization of EV markers is Western blotting^20^ typically requiring >10 μg of protein for the detection of a single marker.^21^ For MSC-EVs, this translates to culture of 2–10 million MSCs, depending on the tissue source, harvesting time, and isolation method used for the MSC-EVs.^22,23^ This implies that the analysis of multiple markers for EV characterization using Western blotting requires a relatively high amount of EV sample input and is time consuming.^21^ A recently developed alternative for EV characterization is a bead-based Western blotting technique called DigiWest.^24^

DigiWest uses a combination of Western blot and a bead-based microarray platform to allow the parallel identification of multiple markers. DigiWest has shown a comparable sensitivity to Western blot, but requires one-hundredth of a traditional Western blotting sample lane for the detection of one marker, displaying its potential use for low protein samples. It has been successfully used to identify proteins in cell lysates.^24^ This study aims to perform EV characterization based on the ISEV guidelines using DigiWest.

To show the potential of the technique, a spectrum of EVs relevant in regenerative medicine was characterized, involving EV-containing samples from different sources and species. Human MSC-EVs isolated from conditioned cell CM^25^ (well characterized by other techniques^26^) were included in this study. Furthermore, EVs derived from CM of pig and dog notochordal cell (NC)-rich tissue, known to contain EVs with regenerative potential^27^ for patients suffering from low back pain due to intervertebral disk degeneration,^28^ were used.

Lastly, EVs isolated from a complex biological fluid (human milk) were incorporated in the analysis. Milk-derived EVs have strong immune modulatory and anti-inflammatory effects.^29^ These characteristics and their ability to be administered orally triggered the interest in the therapeutic use of milk-EVs in chronic and degenerative inflammatory disease.^30^

## Methods

### DigiWest technology

**FIG. 1.**
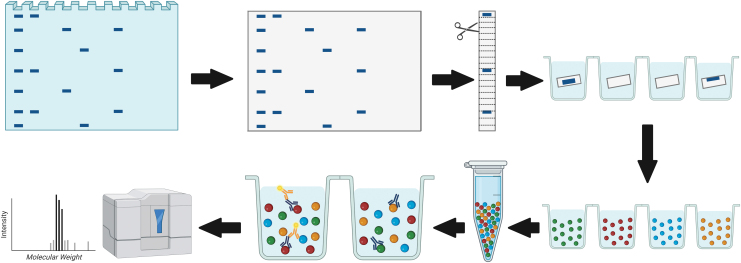
Schematic representation of DigiWest technology workflow. Comparable with standard Western blotting, proteins are separated by gel electrophoresis and transferred to a blotting membrane. The membrane is cut horizontally into strips representing molecular weight fractions. After protein solubilization, distinct color-coded Neutravidin-coated Luminex bead sets are added. The colored Luminex beads are pooled and an aliquot of the bead pool is used for incubation with primary antibodies. After the addition of secondary antibody, the sample is read on a Luminex instrument. The obtained signals are visualized as peaks of fluorescence when plotted against the molecular weight fractions. Color images are available online.

DigiWest was performed as described previously^24^ (Fig. 1). In brief, gel electrophoresis and Western blotting were performed using the NuPAGE system (Life Technologies) with a 4–12% Bis-Tris gel and polyvinylidene fluoride membranes. All proteins were biotinylated on the blot membrane and blots were washed in phosphate buffered saline (PBS) containing 0.1% Tween-20 (PBST) and dried overnight. Each lane was cut into 96 stripes of 0.5 mm and sorted into a 96-well plate (Greiner Bio-One).

After protein elution in 10 μL of 8 M urea in 100 mM Tris-HCl (pH 9.5) including 1% Triton-X100, neutravidin coated color-coded MagPlex beads (Luminex) were added. After overnight coupling, leftover coupling sites were blocked with deactivated NHS-PEG12-biotin (500 μM, 1 h). The DigiWest beads were pooled and the original sample lane was reconstructed by reassigning the color IDs of the MagPlex beads to the molecular weight (MW) fraction.

DigiWest beads were blocked in assay buffer (ELISA blocking reagent) supplemented with 0.2% milk powder, 0.05% Tween-20, and 0.02% sodium azide in a 96-well plate (Corning). Beads were incubated in 30 μL primary antibody (Supplementary Table S1) at 15°C overnight. After washing twice with PBST, R-phycoerythrin-conjugated secondary antibody (Supplementary Table S2) was added for 1 h at 23°C. Then, beads were washed twice with PBST and readout was performed on a Luminex FlexMAP 3D instrument.

For peak identification and quantification of the antibody-specific signals, the DigiWest analysis tool^24^ was employed. This tool uses the 96 values for each initial lane obtained from the Luminex measurements on the 96 MW fractions and calculates a baseline using the bead background (e.g., empty beads measured on the Luminex) and secondary antibody-specific signal (e.g., bead incubation with species-specific antibody only). After subtraction of the background, the tool identifies the peaks at the appropriate MW and integrates the peaks. The reported values present the peak-specific fluorescence intensity (accumulated fluorescence intensity [AFI]).

## Experiment Design

**FIG. 2.**
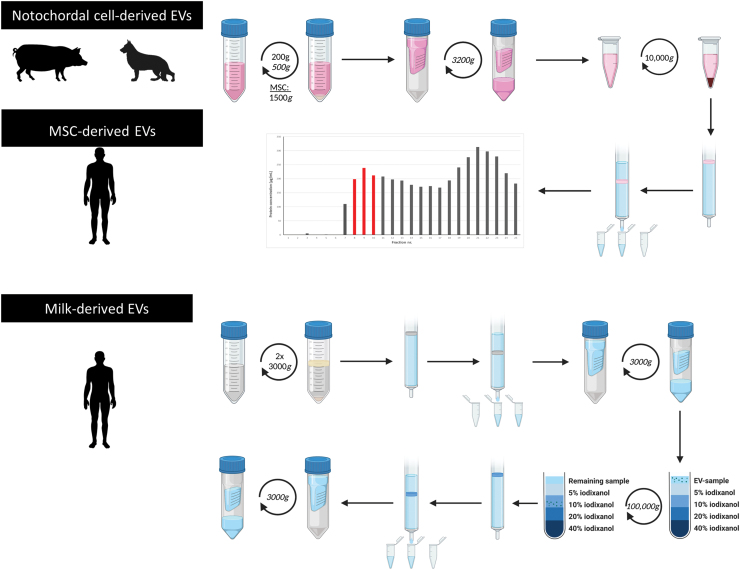
Schematic representation of the EV isolation procedure for the different sample types. EVs were isolated from cell-conditioned medium of human MSCs, tissue-conditioned medium of pig and dog NCs, and a biological fluid (human milk). NC-derived EVs and MSC-derived EVs were isolated with a combination of differential centrifugation and SEC. After SEC, EV containing fractions (8–10) were pooled based on protein content. Milk-derived EVs were isolated using differential centrifugation, SEC, and density gradient centrifugation. *n* = 3 for human and dog samples, *n* = 6 for pig samples. EVs, extracellular vesicles; MSCs, mesenchymal stromal cells; NCs, notochordal cells; SEC, size exclusion chromatography. Color images are available online.

EVs were isolated from CM (MSC-EVs and NC-EVs) and a complex biological fluid (milk-EVs) (Fig. 2).

### MSC culture, characterization, and generation of human MSC-derived EVs

Bone marrow-derived MSCs were derived by the UMC Utrecht Gene and Cell Therapy facility from three independent production batches from anonymous human donors upon written consent. MSCs were expanded in Dulbecco's Alpha MEM (ThermoFisher Scientific) supplemented with 10% fetal calf serum (FCS; Biowest), 100 U/mL penicillin, 100 μg/mL streptomycin, 0.1 μg/mL primocin, 200 μM l-ascorbic acid (Sigma), and 1 ng/mL basic fibroblast growth factor (ThermoFisher Scientific).^31^

The characterization of these MSCs with regard to the CD marker profile and their ability to differentiate into different lineages has been reported previously.^32^ To generate EVs, MSCs were grown to 80% confluency, whereafter the expansion medium was replaced for medium without FCS and primocin. After 24 h, CM was harvested and processed further for EV isolation.

### Generation of pig and dog NC-derived EVs

EVs were isolated from healthy NC-rich intervertebral disks (Thompson grade I^33^) of six pig and three dog donors. Whole pig spines (3 months old) were collected from the local slaughterhouse in accordance with national regulations. Dog spines (18 months old) were collected from mixed breed dogs that were euthanized in unrelated research studies (AVD1080020173964). Intervertebral discs were opened under sterile conditions and NC-rich nucleus pulposus (NP) tissue was collected. NC-rich NP tissue and NCs, isolated as described previously,^34^ were employed as positive control samples for DigiWest.

For NC-EV generation, CM was generated by culturing NC-rich tissue for 4 days (1 g tissue/30 mL medium) in HgDMEM+Glutamax (31966; Gibco) with 1% Penicillin-Streptomycin (15140122; Gibco) at 37°C, 5% CO_2_, and 5% O_2_ as described previously.^35^ After 4 days, the CM was filtered through a 70 μm cell strainer and thereafter processed for EV isolation.

### EV isolation from NC- and MSC-CM

EVs present in CM from NCs and MSCs were purified through differential centrifugation and size exclusion chromatography (SEC) (Fig. 2) as described previously.^27^ NC-CM was centrifuged twice sequentially at 200 and 500 *g* (10 min, 4°C) to remove cells. MSC-CM was centrifuged once at 1500 *g* for 15 min. The supernatant was concentrated 15 times (MSC-CM and dog NC-CM) or 5 times (pig NC-CM) using a 3 kDa Amicon Ultra-15 centrifugal filter tube at 3214 *g* at 4°C for the required time, until ∼500 μL was left in the filter (2–5 h).

All substances with an MW >3 kDa were resuspended in 1 mL PBS (Gibco; 10010023). To remove remaining cellular debris and apoptotic bodies, the concentrated CM was centrifuged at 10,000 *g* (4°C, 35 min). The supernatant was aliquoted and stored at −80°C until further use.

For SEC, qEV SEC-columns (iZON Science; 1 mL sample/column) were calibrated and eluted with PBS (Gibco; 10010023). For each sample, 25 fractions of 0.5 mL were collected per qEV column (MSC-derived EVs: 8 columns; dog NC-derived EVs: 1 column; pig NC-derived EVs: 6 columns). The protein concentration of each fraction was determined at 280 nm (DeNovix; DS-11).

Based on the expected EV sizes and the measured protein concentrations, three fractions with the most EVs (between fractions 7 and 11) were pooled per donor, yielding 1.5 mL of EV-enriched sample. The pooled samples were topped up with PBS in SW41 tubes and centrifuged at 100,000 *g* (65 min, 4°C; Beckman Coulter). The 100,000 *g* pellets of the pooled-EV fractions were resuspended in a maximum of 35 μL PBS per donor and stored at −80°C until further use.

Lastly, to explore the possibility of DigiWest to analyze multiple markers on low protein samples and to show that the SEC fractions selected for NC-EVs indeed contained EVs, for one pig donor (3 months old), all collected SEC fractions were pooled in sets of three (fraction 2–4, 5–7, 8–10, 11–13, 14–16, 17–19, 20–22, and 23–25) to analyze the presence of the EV markers.

### Human milk collection

Milk samples were donated by healthy mothers (mean age of 33 ± 2.3 years) who gave birth through vaginal delivery between 2015 and 2018 and were at a lactational stage of 3 to 7 months (with an average of 4.7 ± 2 months). The Medical Research Involving Human Subjects Act did not apply according to the Hospital Medical Research Ethics Committee. Informed consent was signed by all donors. In brief, mothers were asked to collect milk by using an electric breast pump. Milk was prevented from cooling down, and within 30 min after collection, it was depleted from cells and fat by two rounds of centrifugation at 3000 *g* (10 min, 22°C; Beckman Coulter Allegra X-12R) as described previously.^29^ Cell and fat-free milk supernatants were stored at −80°C until further processing.

### EV isolation from human milk

Milk-EVs were isolated from 6 mL of cell and fat-free milk supernatant according to a published protocol^36^ with minor modifications (Fig. 2). Once thawed, 2 mL of supernatant was loaded into a 10 mL syringe (BD Biosciences) stacked with 10 mL Sepharose CL-2B (GE Healthcare) on top of a 20 μm pore size nylon net (NY2002500; Merck Millipore). A total of three columns were used for each supernatant. Fractions of 1 mL eluate were collected.

Three milliliters EV-containing eluates (eluates 4–6) were concentrated to 1 mL using a Amicon Ultra-2 Centrifugal Filter Unit (UFC201024; Merck Millipore) centrifuged at 3000 *g* for 10–20 min at 4°C. One milliliter concentrated sample was retrieved by upside-down centrifugation at 1000 *g* for 2 min at 4°C and placed on top of a Optiprep (Axis-Shield) density gradient. Discontinuous iodixanol gradients were prepared by layering 4 mL of 40%, 4 mL of 20%, 4 mL of 10%, and 3.5 mL of 5% iodixanol in a 16.8 mL open top polyallomer tube (Beckman Coulter).

Gradient centrifugation was performed at 100,000 *g* for 18 h at 4°C using a SW32.1 Ti rotor (Beckman Coulter). Fractions of 1 mL were collected and EV-rich fractions 9 and 10, corresponding to a density of 1.01–1.18 g/mL, were pooled. Pooled fractions underwent SEC with Sepharose CL-2B and concentration in Amicon Ultra-2 Centrifugal Filter Unit at 3000 *g* at 4°C until a final sample volume of 100 μL was reached. EV samples were stored at −80°C until further use.

### Protein quantification of human milk-EVs

Milk-EV samples were quantified by Qubit Protein assay kit (ThermoFisher Scientific) according to manufacturer's instructions. In brief, 5 μL of milk-EVs (lysing condition sodium dodecyl sulfate [SDS] 0.2%) was incubated with 195 μL of Qubit Working solution at room temperature in the dark for 15 min. Protein concentration was measured by using the Qubit Fluorometer 3.0 (ThermoFisher Scientific).

### Lysis and quality control DigiWest

EV and positive control (MSCs, NCs, and NC-rich NP tissue) samples were diluted 1:2 in 2 × lysis buffer containing 4% lithium dodecyl sulfate, 50 mM of dithiothreitol (DTT), cOmplete™ protease inhibitor and PhosSTOP™ phosphatase inhibitor (Roche), denatured at 95°C for 10 min, and homogenized by centrifugation through a QIAshredder device (Qiagen). One microliter lysate was diluted in loading buffer (212 mM Tris, 282 mM Tris base, 1.01 mM ethylenediaminetetraacetic acid, and 50 mM DTT, 10% glycerol, 0.22 mM Coomassie brilliant blue), and applied to SDS-PAGE using a NuPAGE system (Life Technologies) with a 4–12% Bis-Tris gel. Protein was determined using a LI-COR Odyssey Classic imaging system.

### Data analysis

For correct signal detection for the markers, peaks shown in the DigiWest analysis tool were assessed for several criteria. First, the minimal value of the peak height had to be at least 50. The peak area, that is, the AFI, had to be at least 100. If these criteria were met, the MW of the peak was matched with the MW reported for the respective antibody. If a shift (≤10 kDa) in MW was observed compared with the predicted MW, it was checked whether this shift was consistent in all samples. If consistent and the band at the corresponding MW was also present in the documentation of the antibody supplier, the signal for this antibody was considered to be specific for the marker.

## Experimental Results

### Samples and protein input for DigiWest analysis

The protein amount loaded for cell and tissue lysates was generally higher (8–10 μg protein) than for EV-samples (Table 1). The latter also depended on the EV source, for example, for MSC-EVs a considerable lower protein yield was obtained after isolation when compared with milk-EVs. Since there were considerable differences in protein yield in EV-samples between donors, either the maximum input of that donor or the maximum protein load for DigiWest (10 μg) was used.

**Table 1.** Overview of the Protein Input Used for DigiWest for All Samples, Shown per Donor

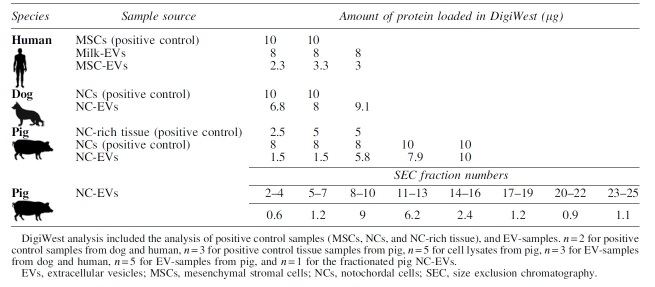

### Antibody optimization for EV characterization with DigiWest

**FIG. 3.**
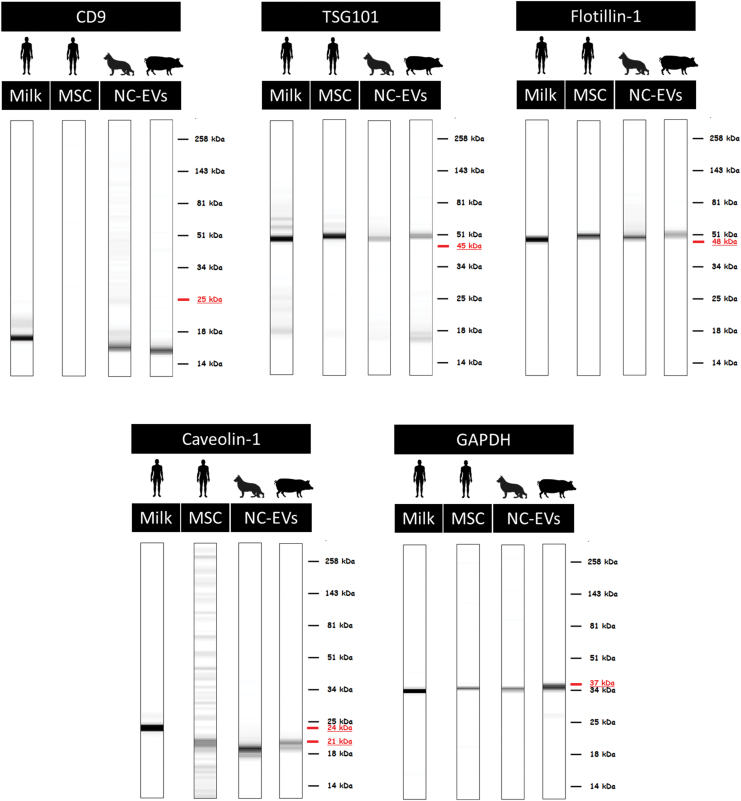
Western blot mimics of five general EV markers in the different species. For humans, EVs samples from milk and MSCs were analyzed. For dog and pig samples, EVs from NCs were analyzed. The *underlined* MW indicates the predicated MW for that marker, based on the antibody information. *n* = 3 for human and dog samples, *n* = 5 for pig samples. MW, molecular weight. Color images are available online.

Based on the ISEV recommended protein markers and the availability of the antibodies, 30 antibodies were selected for the characterization (Supplementary Table S1). Of this panel, 15 antibodies were successfully optimized for all three species (Table 2 and Supplementary Fig. S1). DigiWest analysis of EVs from different species and sources revealed a remarkable overlap in the presence of nine general EV markers, that is, flotillin-1, TSG101, caveolin-1, HSP70, HSPA8, annexin II, glyceraldehyde 3-phophate dehydrogenase (GAPDH), fibronectin, and enolase-1 (Table 1 and Fig. 3).

**Table 2.** List of Optimized Antibodies for Extracellular Vesicles Samples of All Three Species

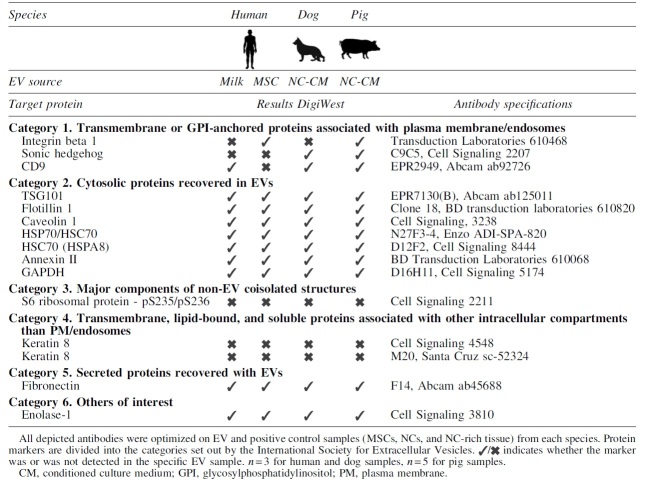

Three of the coisolated proteins, keratin 8, keratin 18, and S6 ribosomal protein, were undetectable in all EV-samples, but present in control samples. Sonic hedgehog (SHH) and integrin beta 1 (ITGB1) were only detected in EV-samples from two species. The tetraspanin CD9 was detected in all species, but not in MSC-EVs.

### DigiWest enables the visualization of data similar to Western blotting

From the digital signals obtained from the DigiWest analysis, data can be visualized resembling traditional Western blots, so called Western blot mimics (Fig. 3). The intensity of the depicted bands in these mimics cannot be compared directly between samples and experiments, it only allows for visualization of the MW of the signal in different samples. In general, the MW of the DigiWest signal corresponded with the predicted MW of the antibody for the different samples and species (e.g., TSG101, flotillin-1, and GAPDH; Fig. 3).

For antibodies that were predicted to detect a protein at multiple MWs because of different isoforms, differences between species/samples were observed. Caveolin-1 had a signal at 24 kDa in human milk-EVs, whereas EVs from other sources (human MSCs, pig and dog NC) only showed a signal at 21 kDa. CD9 showed a band at a much lower MW than expected. However, since this shift was consistent between all samples and a clear peak was observed in the DigiWest analysis tool at this height, the signal was considered specific. Altogether, these results indicate that DigiWest is a robust method facilitating the detection of multiple EV markers in low protein containing EV samples, independent from tissue source and species.

### DigiWest allows for simultaneous detection of multiple proteins in a single EV sample

The optimized antibody panel was used to analyze the SEC fractions from pig NC-CM to confirm the presence of EVs in the fractions selected for the previous analysis and to determine the potential of DigiWest for low protein samples. The protein quantities in SEC fractions ranged from 0.6 to 9 μg, with the first three fractions containing the least amount of protein (Table 1). Owing to too low protein amounts (<0.9 μg protein), fractions 2–4 and 23–25 were excluded from the analysis. All other fractions contained sufficient protein (>0.9 μg) to obtain a steady DigiWest signal.

**FIG. 4.**
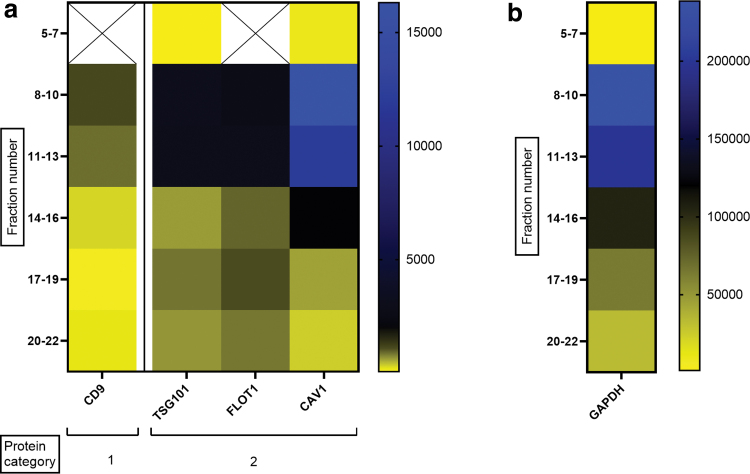
Heat map of five markers in the SEC fractions of pig NC-CM. **(a)** Profile of the AFI of four EV markers within the pooled SEC fractions. The X-mark indicates that marker was too low for peak detection in the SEC fractions. **(b)** Profile of the AFI of GAPDH within the SEC fractions. *n* = 1. AFI, accumulated fluorescence intensity; GAPDH, glyceraldehyde 3-phophate dehydrogenase. Color images are available online.

An enrichment in the EV markers CD9, TSG101, flotillin-1, and caveolin-1 was detected in fractions 8–10 and 11–13 (Fig. 4a). A similar pattern was obtained for GAPDH (Fig. 4b). In contrast to the previously analyzed samples, in the SEC fractions from pig NC-CM a signal was obtained for S6 ribosomal protein in fractions 8–13 (Supplementary Fig. S2), suggesting that this protein had been coisolated. Altogether, these results indicate that 0.9 μg protein input was sufficient for analyzing multiple EV markers in the EV-samples used in this study. Moreover, SEC fractions 8–13 appeared to contain the most EVs according to the expression profile of general EV markers, which confirms that the right fractions were selected for DigiWest analysis of NC-EVs.

### DigiWest shows overlap in identified markers with mass spectrometry

Since this is the first reported use of DigiWest for EV characterization, we compared the obtained results with published proteomics mass spectrometry data of human milk and bone marrow-derived MSC-EVs.^29,37,38^ For milk-derived EVs, the 13 proteins detected with DigiWest were, apart from caveolin 1, all detected previously by mass spectrometry analysis (Table 3).^29,37^ Of the 12 proteins detected by DigiWest analysis of MSC-EVs, all were previously identified in proteomics.^38^ In conclusion, targeted proteomic analysis based on DigiWest shows a reasonable good overlap with mass spectrometry analysis.

**Table 3.** Comparison Between the Results Obtained for the Analyzed Protein Markers with the DigiWest Technology and Mass Spectrometry for Human Milk-Derived Extracellular Vesicles and Human Bone Derived-Mesenchymal Stromal Cell Extracellular Vesicles

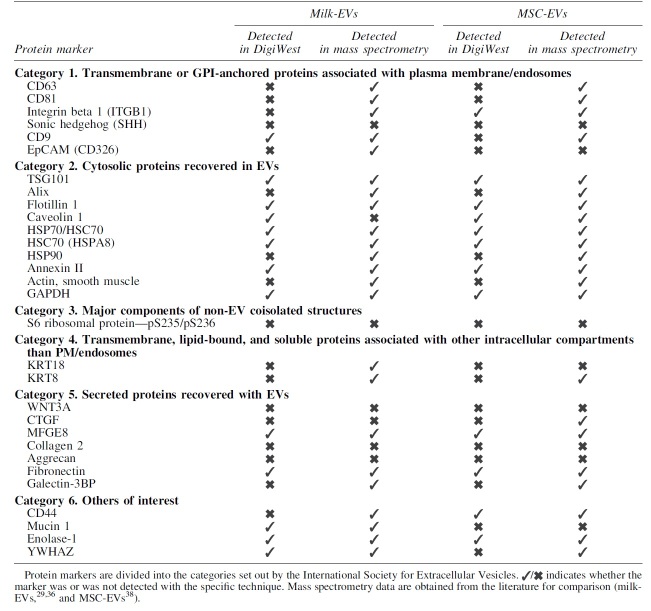

## Discussion

Characterization of EV preparations is essential when working with EVs in either a research or (pre-)clinical setting. The ISEV guidelines recommend the analysis of multiple protein markers in EV samples to demonstrate the presence of EVs and to get an indication on coisolated proteins. This study is the first to report the successful use of a multiplex bead-based Western blotting platform (DigiWest)^24^ for EV characterization of EVs isolated from CM and complex biological fluids from different sources and species. The optimized antibody panel mainly entails general EV markers,^39^ making the created panel presumably convenient for the characterization of EVs from other sources than those reported here.

The panel selected is dynamic and can be modified on the EV type or the specific characterization that is required. DigiWest is restricted by the availability of antibodies, but with the availability of many antibodies for Western blotting, a wide range of markers is possible.

Three of the optimized markers could only be used in specific species (ITGB1 and SHH) or EV sources (CD9). For ITGB1 and CD9, the absence of detection in, respectively, human milk-EVs and MSC-EV is considered to be related either to the sensitivity of the DigiWest technology or to a relatively low starting protein quantity resulting in undetectable levels. ITGB1 was previously detected in milk-EVs^29,36^ and CD9 has been shown in MSC-EVs^38^ with mass spectrometry, a methodology considered to be more sensitive than Western blot-based techniques^40^ such as DigiWest.

SHH is expressed during embryonic development in the notochord^41^ and, therefore, also in the cells that descent from the notochord, the NCs^42,43^ explaining SHH being NC-EV associated. Although SHH is known to be expressed during breast development, its expression in adult healthy mammae tissue is considered low.^44^ The absence of SHH in human milk-EVs and MSCs may thus be tissue dependent.

In addition to showing the presence of EVs in the sample, EVs can be assessed by studying possible coisolated proteins.^19^ Three of such proteins, that is, keratin 8, keratin 18, and S6 ribosomal protein, were successfully validated using positive control (tissue/cell) samples but were undetectable in the majority of the studied EV samples. In the SEC fractions from the pig NC-EV donor studied, S6 ribosomal protein was detected, indicating the presence of ribosomal proteins. This observation implies impurity of the sample and that DigiWest can be used to assess coisolated proteins in EV samples.

Traditional Western blotting requires ∼10 μg of protein for the identification of an individual protein marker, which can be a limitation for EV analysis. DigiWest commonly employs between 5 and 20 μg protein. It must be noted, however, that with DigiWest, this protein input can be used to test >100 antibodies,^24^ whereas for conventional Western blotting, this protein input is used per antibody. We here show that a total of nine markers were successfully analyzed in pig NC-EV samples containing 0.9–9 μg protein input. Multiple markers in samples with a low protein content (≥1 μg) can thus be determined, whereas the minimum amount of protein required for DigiWest technology may, however, depend on the source and complexity of the samples.^24^

Mass spectrometry analysis has gained interest in determining EV cargo because of its sensitivity and specificity compared with other techniques.^40^ In retrospect analysis with the mass spectrometry analysis of MSC-EVs and milk-EVs described in the literature,^29,37,38^ there was an overlap for 12 proteins with those identified through DigiWest. DigiWest failed in the majority of samples to demonstrate the presence of tetraspanins, proteins that were detected in a large number of milk-EV donors,^29,36^ and MSC-EV donors^38^ through proteomic analysis (mass spectrometry).

This discrepancy can be explained by the inability of this study to successfully optimize antibodies for these markers, as these proteins were also not detected in positive control samples. This clearly shows a major limitation of the DigiWest technique that, similar to Western blotting and other techniques using antibody-based detection, still requires a working antibody for that species limiting its application.^24^

Interestingly, DigiWest identified caveolin 1 in human milk-EVs, which was not previously detected with mass spectrometry. Although caveolin 1 has not been described in milk-EVs, it is expressed in healthy mammary cells^45^ and has an important role in EV biogenesis.^46^ A possible explanation for this discrepancy between DigiWest and mass spectrometry might be the structure of caveolins. Caveolin 1 contains a highly hydrophobic central domain that forms a hairpin structure in the membrane,^47^ which can be challenging for mass spectrometry analysis. Proteolytic hydrolysis of the proteins into peptides before mass spectrometry is generally performed by using trypsin. However, membrane proteins typically contain few cleavage sides for trypsin.^48^ The use of trypsin for the identification of membrane proteins, such as caveolin 1, can limit the generation of peptides of suitable size for mass spectrometry identification. Therefore, the absence of caveolin 1 in human milk-EVs with mass spectrometry might be explained by the use of trypsin as protease.

## Conclusions

This study shows that DigiWest is a robust method for the detection of multiple protein markers in EV samples, independent from source and species. The optimized panel entails general EV markers validated for the application in human, dog, and pig derived EV-samples and may, therefore, also be relevant for the characterization of EV samples from alternative sources. Furthermore, DigiWest has been successfully used in EV samples with a low protein content, showing the applicability of this technique for such sample types.

Although DigiWest shows a good overlap with previously obtained mass spectrometry data, DigiWest is a targeted technique for EV characterization rather than an unbiased screening of EV content. With the availability of many antibodies for Western blotting, the antibody panel can easily be tailored to individual EV sources and research questions. Altogether, this study demonstrates the relevance of the DigiWest technique for the characterization of multiple EV types in the regenerative medicine field.

## Supplementary Material

Supplemental data

Supplemental data

Supplemental data

Supplemental data
