# Supplementary material for: A Combined Western and Bead-Based Multiplex Platform to Characterize Extracellular Vesicles
Source: Tissue Eng Part C Methods. 2023 Nov 6;29(11):493–504. doi: 10.1089/ten.tec.2023.0056 (PMC10654656; doi:10.1089/ten.tec.2023.0056)
Supplement: Supplemental data [file Suppl_TableS1.docx]

**Supplementary table 1. Primary antibodies tested on EV-samples with DigiWest**

| **Category 1. Transmembrane or GPI-anchored proteins associated to plasma membrane/endosomes** | | | | | |
| --- | --- | --- | --- | --- | --- |
| *Subcategory* | *Target protein* | *Clone* | *Manufacturer* | *Concentration used in DigiWest (µg/mL)* | *If concentration is unknown, dilution used in DigiWest* |
| 1a. | CD63 | TS63 | Abcam, ab59479 | 5 | N/A |
| 1a. | CD81 | 5A6 | BioLegend, 349502 | 2.5 | N/A |
| 1a. | Integrin beta 1 (ITGB1) | - | Abcam,  ab7168 | N/A | 1:200 |
| 1a. | Integrin beta 1 (ITGB1) | - | Transduction Laboratories,  610468 | 0.25 | N/A |
| 1a. | Sonic Hedgehog (SHH) | C9C5 | Cell Signaling, 2207 | N/A | 1:200 |
| 1a. | Sonic Hedgehog (SHH) | - | R&D, AF445 | 4 | N/A |
| 1b. | CD9 | HI9a | BioLegend, 312102 | 2.5 | N/A |
| 1b. | CD9 | EPR2949 | Abcam, ab92726 | 0.5 | N/A |
| 1b. | EpCAM (CD326) | - | Cell Signaling, 3599 | N/A | 1:200 |
| **Category 2. Cytosolic proteins recovered in EVs** | | | | | |
| 2a. | TSG101 | C-2 | Santa Cruz, sc-7964 | 1 | N/A |
| 2a. | TSG101 | EPR7130(B) | Abcam, ab125011 | 0.23 | N/A |
| 2a. | Alix | 3A9 | Cell Signaling, 2171 | N/A | 1:200 |
| 2a. | Flotillin 1 | 18 | BD transduction laboratories, 610820 | 1.25 | N/A |
| 2a. | Caveolin 1 | 2297 | BD Transduction Laboratories, 610406 | 1.25 | N/A |
| 2a. | Caveolin 1 | - | Cell Signaling, 3238 | N/A | 1:200 |
| 2b. | HSP70 | - | Cell Signaling, 4876 | N/A | 1:200 |
| 2b. | HSP70/HSC70 | N27F3-4 | Enzo, ADI-SPA-820 | N/A | 1:200 |
| 2b. | HSC70 (HSPA8) | D12F2 | Cell Signaling, 8444 | N/A | 1:200 |
| 2b. | HSP90 | S88 | Abcam, ab1429-50 | 4 | N/A |
| 2b. | HSP90 a/b | AC88 | Santa Cruz, sc-59577 | 0.5 | N/A |
| 2b. | Annexin II | - | BD Transduction Laboratories, 610068 | 0.25 | N/A |
| 2b. | Actin, Smooth Muscle | 1A4 | Biogenex, MU128-UC | N/A | 1:50 |
| 2b. | Alpha-Smooth Muscle Actin | - | Cell Signaling, 14968 | N/A | 1:200 |
| 2b. | Actin beta | AC-15 | Sigma, A1978 | 5 | N/A |
| 2b. | GAPDH | - | Abcam, ab9484 | N/A | 1:200 |
| 2b. | GAPDH | D16H11 | Cell Signaling, 5174 | N/A | 1:200 |
| **Category 3. Major components of non-EV co-isolated structures** | | | | | |
| 3b. | S6 ribosomal protein - pS235/pS236 | - | Cell Signaling, 2211 | N/A | 1:200 |
| **Category 4. Transmembrane, lipid-bound and soluble proteins associated with other intracellular compartments than PM/endosomes** | | | | | |
| 4d. | KRT18 | - | Cell Signaling, 4548 | N/A | 1:200 |
| 4d. | KRT8 | C-43 | Invitrogen, MA1-19037 | N/A | 1:200 |
| 4d. | KRT8 | M20 | Santa Cruz, sc-52324 | 0.25 | N/A |
| **Category 5. Secreted proteins recovered with EVs** | | | | | |
| 5a. | WNT3A | - | Millipore, 09-162 | 2.5 | N/A |
| 5a. | CTGF | - | Abcam, ab6992 | 2.5 | N/A |
| 5b. | MFGE8 | - | Sigma, HPA002807 | N/A | 1:200 |
| 5b. | Collagen 2 | II-4C11 | MP Biomedicals, 8631711 | 4.98 | N/A |
| 5b. | Collagen 2 | II-II6B3 | DSHB, II-II6B3 | N/A | 1:200 |
| 5b. | Aggrecan G1-IGD-G2 Domains | 179509 | R&D, MAB1220 | N/A |  |
| 5b. | Fibronectin | F14 | Abcam, ab45688 | N/A | 1:200 |
| 5b. | Galectin-3BP | - | R&D, AF2226 | 1 | N/A |
| **Category 6. Others of interest** | | | | | |
| N/A | CD44 | EPR1013Y | Epitomics, 1998-1 | N/A | 1:1000 |
| N/A | Mucin 1 | VU4H5 | Santa Cruz, sc-7313 | 1 | N/A |
| N/A | Enolase-1 | - | Cell Signaling, 3810 | N/A | 1:200 |
| N/A | YWHAZ | D7H5 | Cell Signaling, 7413 | N/A | 1:200 |
