# Supplementary material for: A Combined Western and Bead-Based Multiplex Platform to Characterize Extracellular Vesicles
Source: Tissue Eng Part C Methods. 2023 Nov 6;29(11):493–504. doi: 10.1089/ten.tec.2023.0056 (PMC10654656; doi:10.1089/ten.tec.2023.0056)
Supplement: Supplemental data [file Suppl_TableS2.docx]

**Supplementary table 2. Secondary antibodies used for the DigiWest technology**

| ***Secondary antibody*** | ***Manufacturer*** | ***Host - target*** | ***Specificity*** |
| --- | --- | --- | --- |
| R-Phycoerythrin AffiniPure F(ab')₂ | Jackson, 711-116-152 | Donkey Anti-Rabbit | IgG |
| R-Phycoerythrin AffiniPure F(ab')₂ | Jackson, 715-116-151 | Donkey Anti-Mouse | IgG |
| R-Phycoerythrin AffiniPure F(ab')₂ | Jackson, 705-116-147 | Donkey Anti-Goat | IgG |
| R-Phycoerythrin Conjugate | Jackson, 016-110-084 | Streptavidin | N/A |
