# Supplementary material for: A Combined Western and Bead-Based Multiplex Platform to Characterize Extracellular Vesicles
Source: Tissue Eng Part C Methods. 2023 Nov 6;29(11):493–504. doi: 10.1089/ten.tec.2023.0056 (PMC10654656; doi:10.1089/ten.tec.2023.0056)
Supplement: Supplemental data [file Suppl_FigureS1.docx]

**Supplementary figure 1. Heat maps of the optimized antibodies for EV samples of all three species.** (a) Profile of the mean Accumulated fluorescence intensity (AFI) of EV markers within category 1 (as set by International Society for Extracellular Vesicles (ISEV).) (b) Profile of the mean AFI of EV markers within category 2. (c) Profile of the mean AFI of EV markers within category 3-6. The X-mark indicates that the marker was undetectable in the sample type. n=3 for human and dog samples, n=5 for pig samples.

**a**

**b**

**c**
