# Supplementary material for: A Combined Western and Bead-Based Multiplex Platform to Characterize Extracellular Vesicles
Source: Tissue Eng Part C Methods. 2023 Nov 6;29(11):493–504. doi: 10.1089/ten.tec.2023.0056 (PMC10654656; doi:10.1089/ten.tec.2023.0056)
Supplement: Supplemental data [file Suppl_FigureS2.docx]

**Supplementary figure 2. Heat map of S6 ribosomal protein - pS235/pS236 in the SEC fractions 8-10 and 11-13 of pig NC-CM and a positive control sample.** Profile of the Accumulated fluorescent intensity (AFI) of within the three samples. n=1; SEC; size-exclusion chromatography

*
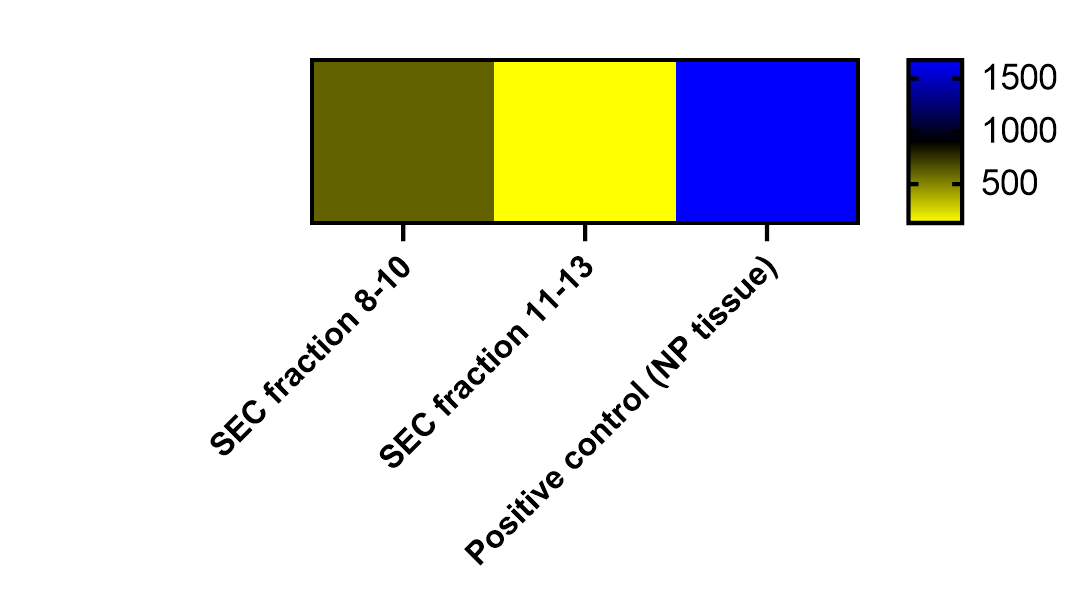
*
